# Perspective Insights of Exosomes in Neurodegenerative Diseases: A Critical Appraisal

**DOI:** 10.3389/fnagi.2017.00317

**Published:** 2017-09-29

**Authors:** Arif Tasleem Jan, Mudasir A. Malik, Safikur Rahman, Hye R. Yeo, Eun J. Lee, Tasduq S. Abdullah, Inho Choi

**Affiliations:** ^1^Department of Medical Biotechnology, Yeungnam University, Gyeongsan, South Korea; ^2^CSIR-Indian Institute of Integrative Medicine, Jammu, India

**Keywords:** diagnostics, drugs, exosomes, neurodegeneration, therapeutics

## Abstract

Exosomes are small membranous entities of endocytic origin. Their production by a wide variety of cells in eukaryotes implicates their roles in the execution of essential processes, especially cellular communication. Exosomes are secreted under both physiological and pathophysiological conditions, and their actions on neighboring and distant cells lead to the modulations of cellular behaviors. They also assist in the delivery of disease causing entities, such as prions, α-syn, and tau, and thus, facilitate spread to non-effected regions and accelerate the progressions of neurodegenerative diseases. The characterization of exosomes, provides information on aberrant processes, and thus, exosome analysis has many clinical applications. Because they are associated with the transport of different cellular entities across the blood-brain barrier (BBB), exosomes might be useful for delivering drugs and other therapeutic molecules to brain. Herein, we review roles played by exosomes in different neurodegenerative diseases, and the possibilities of using them as diagnostic biomarkers of disease progression, drug delivery vehicles and in gene therapy.

## Introduction

Exosomes were first considered to be vesicles produced by mature blood reticulocytes and secreted into extracellular milieu with topically expressed transferrin receptors (Harding and Stahl, 1983; Johnstone et al., 1987). Unlike microvesicles, which arise from outward budding of plasma membrane, apoptotic bodies (fragments of dying cells), and ectosomes, exosomes are produced by the fusion of multivesicular bodies (MVBs) with membranes. Exosomes are homogenous and smaller than other extracellular vesicles, with sizes ranging between 30–100 nm (Raposo and Stoorvogel, 2013; Keerthikumar et al., 2015). They are secreted by reticulocytes, mesenchymal cells, neurons, fibroblasts, epithelial cells, endothelial cells, platelets, allophycocyanins (APCs), tumor cells and other cells (Bobrie et al., 2011; Colombo et al., 2014), and are present in bronchioalveolar lavage, synovial fluid, urine, bile, breast milk, serum and other body fluids (Théry et al., 2009; Vlassov et al., 2012) because they display specific cell-derived components on their surfaces, exosomes with different cellular backgrounds possess same set of molecules, such as, enzymes, nucleic acids, cytokines and bioactive compounds. Following release, they are uptaken by surrounding cells or transported systemically in cerebrospinal fluid (CSF; Grapp et al., 2013; Gui et al., 2015), breast milk (Zonneveld et al., 2014), blood (Baranyai et al., 2015), urine (Royo et al., 2016) and in other biological fluids to provide a means of cell-to-cell communication (Bang and Thum, 2012). Accordingly, exosomes influence physiological and pathobiological conditions.

Exosome discovery has exhibited enormous growth over the past three decades. Once known primarily for their role in eliminating excessive cellular proteins and undesirable molecules, exosomes are now known to be required for many physiological processes, such as, the maintenance of normal physiological functions and cell-to-cell communication, and to play important roles in the progression of diseases, such as, cancer and neurodegenerative diseases. Their involvement in neurodegenerative disease progression are attributed to their abilities to transfer biomolecules and pathogenic entities across biological barriers (Candelario and Steindler, 2014; Thompson et al., 2016). Furthermore, their abilities to transport proteins and nucleic acids (siRNA, miRNA) have been exploited for the delivery of drugs and other encapsidated biomolecules. Here, we describe the roles of exosomes in the progressions of neurogenerative disorders, their diagnostic roles as neurodegeneration markers, and their therapeutic applications for the treatment of diseases and for gene therapy.

## Exosome Compositions

Technological advances have resulted in new ways of isolating exosomes by centrifugation, immunocapture and by using microfluidic methods, based on size, shape, density and surface marker expression. Although no consensus has been reached regarding isolation methods, immunoaffinity capture (IAC), chromatography, density gradient centrifugation and ultracentrifugation are the methods more commonly used (Skog et al., 2008). Exosome compositions are dictated by the functional states of cells (Bang and Thum, 2012), though their compositions are largely dependent on their origins (Cosme et al., 2013). Analyses of their compositions by Western blotting (Raposo et al., 1996), Fluorescence Activated Cell Sorting (FACS; Clayton et al., 2001) and mass spectrometry have revealed the presence of endosome associated proteins (Alix, TSG101), a series of tetraspanins (CD9, CD26, CD53, CD63, CD81, CD82), heat shock proteins (HsP70, Hsp90), cytoskeletal elements (tubulin, actin), clathrin, follitin-1, annexins, lysosomal protein (Lamp2b), RAB proteins, intercellular adhesion molecule-1 (ICAM-1), major histocompatibility complexes (MHCs), and co-stimulatory molecules of T-cells, like CD86 (Théry et al., 2002; Keller et al., 2006; Table 1). Proteomic analysis of exosomes also revealed the presence of cell surface anchored sheddases (e.g., ADAM, a disintegrin and metalloproteinase) and cell surface and soluble matrix metalloproteinases (MMPs; Théry et al., 2002). In addition to their extracellular matrix (ECM) remodeling roles, MMPs are critical in cellular interaction, proteosomal processing of exosomal contents and in the intercellular communication. Additionally, protein components, such as, peroxidases and pyruvate kinases, have also been reported in exosomes of enterocyte and human dendritic cells (Théry et al., 2002).

**Table 1:** Table summarizing information on exosomal components.

Constituents	Type	Function
*Alix, TSG101, Rab proteins (Rab11, Rab27b), VAMP7*	MVB formation	Proteins involved in multivesicular (MVB) biogenesis
*CD9, CD26, CD53, CD63,CD81, CD82*	Tetraspannins	Morphogenesis, fission and fusion processes
*Hsc70, Hsp 90*	Heat-shock proteins	Inherent characteristic of exosomes
*GTPases, clathrin and flotillin*	Membrane transport and fusion proteins	Membrane transport and fusion proteins
*Actin, Tubulin and Annexins*	Cytoskeletal elements	Structural
*Metabolic enzymes (peroxidases, lipid and Pyruvate kinases, etc.)*	Metabolism	Regulation of metabolic activity
*mRNA, miRNA, SnRNA*	Genetic material	Gene expression regulation
*Ceramides, phosphatidylserine, diacylglyceride, cholesterol, Phosphatidylcholine, sphingomyelin, etc*.	Phospholipids and cholesterol	Required for signaling and fusion to cell membrane
*MHC molecules, ICAM-1, CD86, P-selectin, immunoglobulin A33*	Immune function	Regulation of immune response (immunosuppressive/immune activation)
*Transmembrane proteins such as αM (DCs), α4β1 (reticulocytes)*	Signaling	Cell specific exosome recognition and characterization

*The table contains information of proteins, nucleic acid and lipid components along with the role they play in exosomes*.

Exosomal membranes are also enriched with lipids, such as, ceramides, phosphatidyl ethanolamine, phosphatidylserine, diacylglyceride, cholesterol, lyso-bis-phosphatidic acid and sphingomyelin (Janas et al., 2015; Haraszti et al., 2016). Although characterization of exosomes depends on the cell type and the protocol used, exosomes have been reported to contain different amounts of DNA (dsDNA, ssDNA, MtDNA), RNA (mRNAs, miRNAs, snRNA, ncRNA) and small cytoplasmic RNAs (van Balkom et al., 2015; Zhang et al., 2015; Kalluri and LeBleu, 2016). Data pertaining to exosomes has been collated in the Exocarta^1^ database, which contains 41,860 proteins, 4956 mRNAs and 1116 miRNAs.

## Exosomes and Neurodegeneration

**Figure 1:**
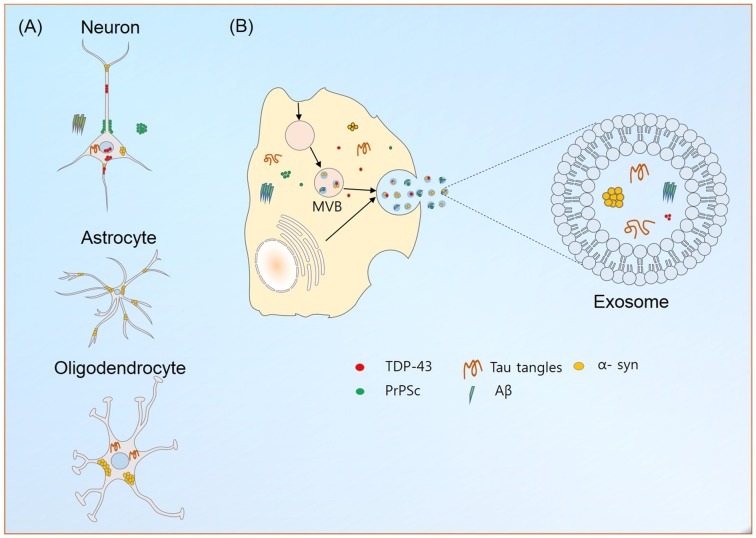
Exosome production and neurodegeneration. **(A)** Localization of aggregated or misfolded proteins credited for their roles in the neurodegeneration in different neuronal cells. **(B)** Exosome production in neuronal cells. The abilities of exosomes to carry disease causing entities to neighboring and distant localized cells contributes to the aggravation of neurological diseases. Similarly, their transport across blood brain barrier (BBB) helps in reducing the possible emergence of different neurological diseases.

Exosome secretion has been reported for a number of cells in the nervous system (Figure 1A). Though exosomes from neurons display GluR2/3 subunit receptor molecules, microglia exosomes contain pro-inflammatory cytokine, IL-β, astrocytes Hsp70, IL-β and synapsin-1 and exosomes from oligodendrocytes contain myelin lipids: cholesterol, galactocerebrosides (Bianco et al., 2009; Kettenmann et al., 2011; Wang et al., 2011). Exosomes have a great effect on cell-to-cell communication, due to: (1) interactions between topical proteins and receptors on target cells; and (2) proteolysis of their cargoes and internalizations of their contents via endocytosis. Furthermore, they allow intercellular communications, via the transport of protein and nucleic acid entities under both normal and diseased states, which suggests exosomes participate in development, cellular function and associated pathologies.

Aggregation of proteins is a hallmark of neurodegenerative diseases, and their accumulations in the CNS hinder mitochondrial and proteosomal functions, axonal transport and synaptic transmission and enhance endoplasmic reticulum stress (Joshi et al., 2014; Yuyama et al., 2015). The ability of exosomes to carry misfolded or aggregated proteins enhances the progression of neurodegenerative diseases (Figure 1B). In line with the prion-like spreading hypothesis (Prusiner, 1982), their implications in the transmission of infectious particles, prions (in Creutzfeldt-Jakob disease, CJD), amyloid precursor protein (APP; in Alzheimer’s disease (AD)), α-synuclein (α-syn; in Parkinson’s disease (PD)) and superoxide dismutase 1 (SOD1; in amyotrophic lateral sclerosis (ALS)) between cells in the nervous system are currently being explored.

### Prion Diseases

Prions are infectious particles that arise due to misfolded or aberrant conformations of proteins (Kupfer et al., 2009; Moore et al., 2009). Given an ability to transfer misfolded states to native forms of similar proteins, the proteopathic state of prions triggers a series of refoldings and aggregations, causing aggregations to oligomers and fibrils (Polymenidou and Cleveland, 2011; Cescatti et al., 2016). Prion-mediated spread of pathophysiological prominence to other cells leads to neurodegeneration. The best example of this mode of transmissible proteinopathy is CJD. The theory of exosome-mediated propagation of prion disease resulted from the studies of Vella et al. (2007, 2008) which showed exosome-associated protein-rich plasma (PrP; encoded by *PRNP*) in the CSF of sheep (Stahl and Prusiner, 1991). Later, Fevrier et al. (2004) reported release of both normal (PrPC) and pathogenic (PrPSc) prion protein in an exosome secreted by PrP-expressing cells. On one side where alteration of the exosome cargo and structure by PrPSc was reported (Bellingham et al., 2012; Coleman et al., 2012), upregulation of exosome secretion was found increasing the effectiveness of PrPSc (Kovacs, 2016).

### Alzheimer’s Disease

AD arises as a result of the extracellular deposition of amyloid-β (Aβ; encoded by *AβPP*) fibrils and of abnormally phosphorylated tau protein (encoded by *MAPT*) in neurons (Iqbal et al., 2010; Murphy and LeVine, 2010; Bloom, 2014). Aβ propagation via exosomes to extracellular milieu was reported by Rajendran et al. (2006) who observed cleavage of APP followed by secretion of β-amyloid in exosomes. Subsequently, secretions of the C-terminal part of APP was observed *in vitro* (Sharples et al., 2008) and *in vivo* (Perez-Gonzalez et al., 2012). Though, exosomes have been reported to induce the formation of neurotoxic oligomers of Aβ (Yuyama et al., 2012; Joshi et al., 2014), they have also been reported to have a neuroprotective role of clearing toxic oligomeric species in exosomal lumen (Dinkins et al., 2014). Impaired Aβ clearance in AD patients and the neuronal and microglial exosome disposal of Aβ (Yuyama et al., 2015) hints at the possibility of a dual clearance mode for Aβ in AD.

Neurofibrillary tangles representing hyper-phosphorylated misfolded tau have been observed in the CSF of AD patients (Šimić et al., 2016). Exosomal secretion of tau is considered critical for the spread of tauopathy to different areas of the AD brain. Contrary to the dependence of tau propagation on the exosomal secretory pathway (Asai et al., 2015), recent studies on the role of exosomes in proteinopathies revealed regulation of Tau secretion by neuronal activities (Wu et al., 2016). Additional work is required to clarify the role played by exosomes in the progression of AD.

### Parkinson’s Disease

Pre-synaptic α-syn (encoded by *SNCA*) protein, which exists as an equilibrium between monomeric and oligomeric states, is a major component of Lewy bodies in PD (Spillantini et al., 1997). Presence of α-Syn in synaptic vesicles suggests its involvement in synaptic vesicle processing (Vargas et al., 2014). α-Syn induced conformational changes facilitates vesicle curvature during their production (Westphal and Chandra, 2013). Lee et al. (2005) reported compartmentalization of α-syn promotes its misfolding, and Grey et al. (2015) reported acceleration of α-syn aggregation in exosomes. Mutagenic and disease like factors favoring fibrillization, a pathogenic process in PD, are thought to propagate in a prion-like process of α-syn misfolding (Olanow and Brundin, 2013). Stuendl et al. (2016) reported the promotion of α-syn aggregation by exosomal loaded α-syn species isolated from CSF of PD patients.

### Amyotrophic Lateral Sclerosis

Misfolding of Cu/Zn SOD1 is a characteristic feature of the familiar and sporadic form of ALS (Bosco et al., 2010). Neuronal secretion of exosomes containing mutant SOD1 was found to transmit pathogenic traits to nearby neurons, such as, the misfolding of SOD1 (Gomes et al., 2007). Exosomes of astrocytic origin loaded with SOD1 show selective toxicity to neurons (Basso et al., 2013) and help establish the exosome-mediated pathogenic behavior of mutant SOD1 in ALS.

TDP-43 is a highly conserved nuclear protein (encoded by *TARDBP*), and is another entity involved in ALS. Its pathogenic mechanisms include cytoplasmic mis-localization, misfolding followed by aggregation, and inclusion formation (Scotter et al., 2015). Dipeptide repeat proteins (DPRs) represent unconventional translation product of C9*ORF*72. Studies indicate an association between TDP-43 and DPRs resulting in the release and transport of exosomes, which supports the notion of exosome-based disease spread (Ding et al., 2015; Westergard et al., 2016).

## Functional Aspects of Exosomes

### Exosomes in Disease Diagnosis

The progressive accumulation of protein aggregates (Brettschneider et al., 2013) and lack of sensitive biomarkers (Poste, 2011) hamper the development of disease-specific treatments for neurodegenerative diseases. Though protein aggregates can be detected in CSF and blood, their extremely low levels limit their usefulnesses as potent biomarkers of neurodegenerative diseases (Blennow and Zetterberg, 2015). Furthermore, low amounts of nucleic acid entities also limit their usefulnesses as biomarkers. Given these limitations, the focus for biomarkers in neurodegenerative diseases has shifted from proteins to the vesicular constituents of biofluids. In addition to the surface localizations of specific proteins, the presence of disease-specific molecular signatures in exosomes makes them strong diagnostic candidates. As they protect their cargoes from degradation (Théry et al., 2002; Chiasserini et al., 2014), screening exosomes in CSF and serum offers a means of identifying their cellular origins and thus provides insights of cellular and pathogenic processes going in CNS. Given evidence of their secretion into CNS (Thompson et al., 2016), it is possible that screening vesicular entities of CSF for vesicle associated membrane protein-2 and enolase (neuron specific markers), integrin α-M (microglia specific marker) and transmembrane protein 132D (oligodendrocyte marker) could be used to detect neurodegenerative diseases. A good example of disease-specific molecular signatures in exosomes is provided by exosomes produced by tumor cells in brain that displays of immunosuppressive and oncogenic factors (van der Vos et al., 2011).

### Exosomes and Therapeutics

The ability of exosomes to cross the blood-brain barrier (BBB) and pass through interstitial fluid into CSF has highlighted their use as drug delivery vehicles targeting the CNS. Systemic administration of exosomes with an siRNA cargo was utilized for drug delivery to the brains of mice (Alvarez-Erviti et al., 2011; Cooper et al., 2014), and the intranasal administration of exosomes loaded with an anti-inflammatory drug was also used for drug delivery to the mouse CNS (Zhuang et al., 2011). Though actual transport routes remain elusive, exosomes have great potential for the delivery of therapeutic RNAs, proteins and small molecules.

#### Delivery of Therapeutic RNA

The abilities of exosomes to transport nucleic acid cargoes (siRNA, miRNA) under physiological and pathological condition have increased interest in exploiting them as drug delivery vehicles and for genetic therapy. This ability is adopted to alter the expressions of genes via the bindings of siRNA or miRNA to complementary mRNA sequences thereby controlling gene expression at the post transcriptional level (Bartel, 2009; Ha et al., 2016). Furthermore, exosomes improve the stabilities of their contents in the systemic circulation and extracellular space, and thus, increase efficacies of delivery. Alvarez-Erviti et al. (2011) demonstrated the therapeutic advantages of exosome based siRNA delivery to mouse brain. They engineered surface localized Lamp2b protein to rabies glycoprotein to assist targeted delivery of therapeutic cargo to brain, and successfully delivered therapeutic GAPDH siRNA to neurons, microglia and oligodendrocytes in the brain. In addition, this study also provided insights regarding their utilization for the delivery of therapeutics to other tissues.

#### Delivery of Therapeutic Proteins

In addition to the delivery of RNA, exosome-based therapeutic protein delivery has also been shown to be beneficial in PD. Characterized by diminished SOD1 and catalase levels (Ambani et al., 1975), reactive oxygen species (ROS) overproduction causes the activation and inflammation of microglia in brain. Therapeutic delivery of catalase loaded into exosomes successfully crossed the BBB and inhibited neurodegeneration (Haney et al., 2015), and improved disease state in PD.

#### Delivery of Small Therapeutic Molecules

Knowing that efficiency of a drug depends on functional transport across the BBB, strategies, such as, delivery as nano-formulations and PEGylated drugs, were commonly employed for targeted drug delivery (Yoshida et al., 1992; Veronese et al., 2002; Agrawal et al., 2014). However, delivery as nano-formulations was discontinued for toxicity reasons and rapid clearance by the mononuclear phagocyte system (MPS), while PEGylated drugs were discontinued because PEGylation reduced target-drug interaction, and thus, affected distribution in the brain. Exosome employment improves small molecule transport across the BBB transport because MPS clearance rates are lower for nano-delivery systems. Sun et al. (2010) adopted an exosomal strategy to deliver curcumin (an anti-inflammatory that reduces the productions of inflammatory cytokines like tumor necrosis factor-α (TNF- α) and interleukin-6 (IL-6)) in brain. Exosomal curcumin delivery increased the solubility, stability and bioavailability of curcumin. Zhuang et al. (2011) followed this by co-administering curcumin and JS1124 (an activator of Stat3 inhibitor) to reduce production of inflammatory cytokines in brain (Sun et al., 2010), and a similar approach was adopted to deliver doxorubicin and paclitaxel across the BBB (Tian et al., 2014; Yang et al., 2015). In addition, Sun et al. (2010) reported that intranasal administration of endosomal GL26 suppressed glioma growth by increasing their microglial uptake. The therapeutic efficiencies of drugs observed in animal models indicate the potential of exosomes for the delivery of drugs across BBB to combat neurodegenerative disorders and brain tumors.

## Exosome Engineering

A large number of studies have been performed on the exosomal delivery of therapeutic molecules, but comparatively little effort has been directed toward engineering exosomes for the target-specific deliveries of therapeutic cargoes. The prerequisite for targeted delivery of therapeutic molecules in different diseases is achieved by surface engineering of targeted peptide or protein on exosomes. Bioengineering of exosomes aimed at achieving correct insertion and avoiding peptide cleavage, results in the expressions of target peptides as fusion to signal peptide of lysosomal associated membrane protein-2b (Lamp-2b). A good example for this phenomena is provided by rabies viral glycoprotein (RVG) and iRGD peptides, which when engineered to exosomes of immature dendritic cells aided targeting of brain and tumor tissues (Alvarez-Erviti et al., 2011; Tian et al., 2014). Bioengineering was found to enhance the cellular uptakes of exosomes, and thereby increase the specificity of treatment in tissues of interest. For example, the surface expression of folate receptor-α (FRα) on choroid plexus epithelial cell derived exosomes was found to direct its cargo to brain parenchymal cells through choroid plexus (Grapp et al., 2013). Target peptide engineering facilitating delivery of drugs to brain tissues on crossing BBB or through choroid plexus, holds considerable promise in terms of overcoming the shortcomings of delivering drugs to brain (Alvarez-Erviti et al., 2011; Grapp et al., 2013). The specific cellular characters of exosomes add advantage for target specific delivery of therapeutic cargo.

## Conclusion

Exosomes are nano-vesicles produced by diverse cell types and perform a multitude of functions in different cellular backgrounds. Although they have been mainly studied in the context of cell-to-cell communication, the surface localizations of specific proteins on exosomes and their disease specific molecular signatures makes them strong candidates for disease diagnosis. Furthermore, their abilities to transport proteins and nucleic acids goes well for their potential exploitation as drugs delivery agents. Because of the range of prospective application offered by exosomes, future work in this area seems necessary; to formulate robust and reproducible extraction protocols, to provide clearer understanding of the pathways involved in the biogenesis of exosomes and the loading of targeted therapeutic moieties, and to identify strategies for engineering exosomes that are capable target-specific drug delivery.

## Author Contributions

IC, TSA and ATJ conceived the idea. ATJ and MAM contributed to writing of the manuscript. ATJ, SR, HRY and EJL proofread the contents for upgradation and prepared the figures.

## Conflict of Interest Statement

The authors declare that the research was conducted in the absence of any commercial or financial relationships that could be construed as a potential conflict of interest.

## Footnotes

^1^www.exocarta.org
